# Supplementary material for: Unobtrusive tracking of interpersonal orienting and distance predicts the subjective quality of social interactions
Source: R Soc Open Sci. 2020 Aug 12;7(8):191815. doi: 10.1098/rsos.191815 (PMC7481680; doi:10.1098/rsos.191815)
Supplement: Supplementary video [file rsos191815supp2.pdf]

# Supplementary material

## Unobtrusive tracking of interpersonal orienting and distance predicts the subjective quality of social interactions

**Juha M. Lahnakoski, Paul A.G. Forbes, Cade McCall, Leonhard Schilbach**

**Royal Society Open Science**

## Supplementary results

### Validation of the motion tracking system

First, we estimated the spatial registration error for realigning the participants into a common coordinate system (**Supplementary Figure 1 A)**. The mean between-sensor error of the location of participant #2 over dyads was generally approximately 3 cm over the entire experiment, with the tail of the distribution (over time) reaching approximately 10 cm. However, some outlier points were observed, where error was >15 cm. Next, we calculated the correlations of motion estimated based on the two Kinect sensors for all 25 joints in the model (**Supplementary Figure 1 B**). For the joints that were visible to both Kinects (indicated by a white background), the correlations are significantly higher than chance. The correlations are particularly high for the head and the upper body (r~0.8). The correlations are reduced gradually (but remain significant) toward the tip of the right hand and are at chance level for the left hand that was usually on the lap of the participants—a pattern that was reversed for the left-handed dyad. The body parts that were occluded by the table or the body of the participants (indicated by a dark gray background) were at chance level, as expected, while the partially occluded middle of the spine (light gray) was still correlated between sensors.

To validate the performance of the Kinects to more traditional wearable sensors, we compared the face orientation data from the head IMUs and Kinects (**Supplementary Figure 1 C** and **D**). Because the reference frame of the IMU angles (magnetic field of the earth) and the Kinects (physical orientation of Kinect #2) are different, we focus here only on the correlation of the angle timecourses rather than direct difference in the angles. The correspondence of the orientations is very high for almost all participants recorded by Kinect #1 (r-values approximately 0.8–0.9). Correlations are reduced for most participants recorded by Kinect #2 (r-values approximately 0.4–0.7) while for a few participants the orientation correspondence was relatively poor (r~0.1–0.3) reducing the overall similarity of the data (r=0.61±0.23 over all participants). The replicability of the acceleration timecourses was lower (r=0.37±0.14) probably due to the short durations of the event-like accelerations compared with the smooth and continuous orientation and location changes, and the differences in sampling rate and sensitivity to small and short accelerations between the sensor types.

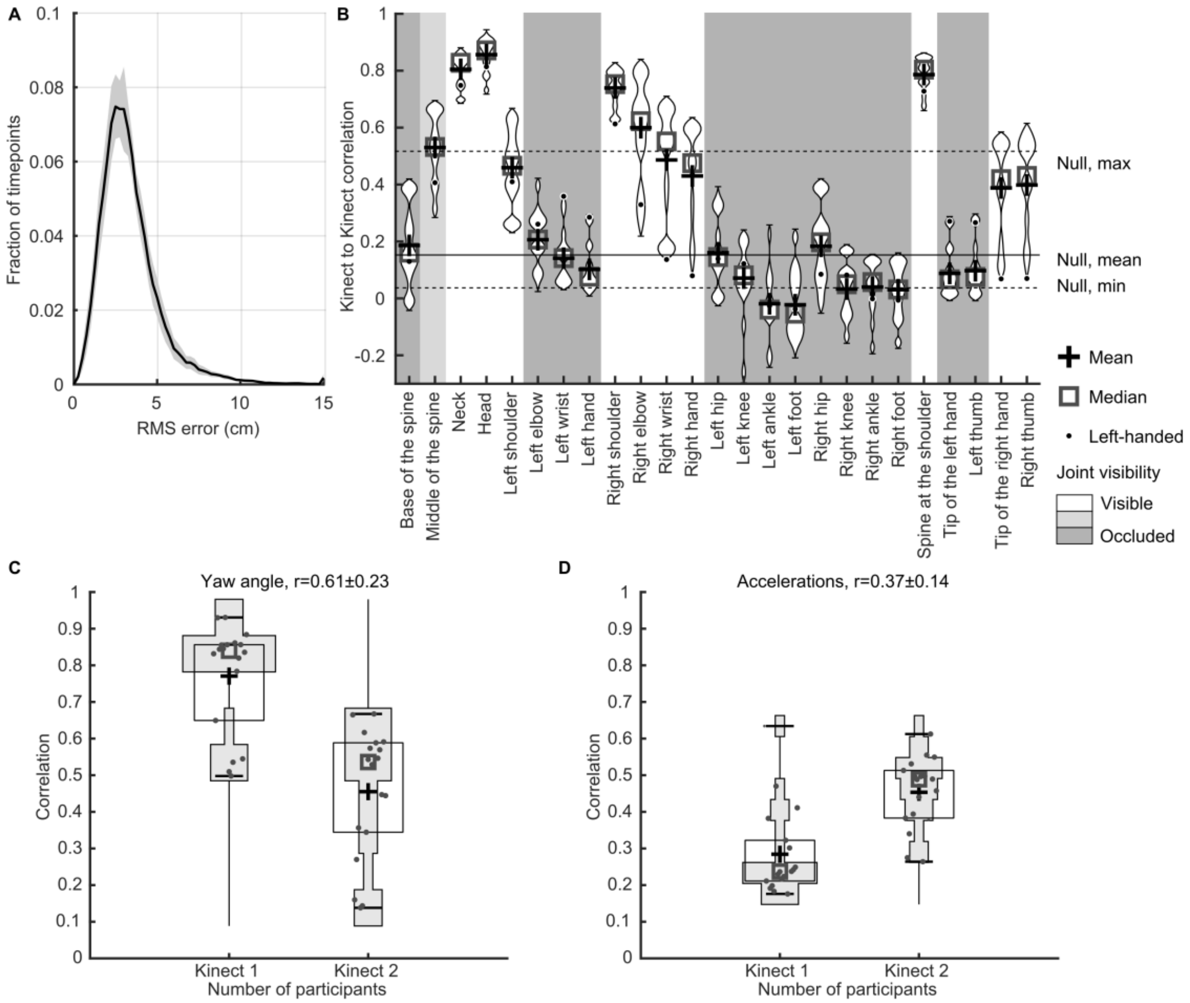


***Supplementary Figure 1: Kinect–Kinect and Kinect–IMU data reliability. A:*** *Distribution of spatial distance (RMS error) between the head location estimates of the two Kinects after registration as a fraction of samples over time. The black line indicates the across-subjects mean distribution of error magnitudes and the shaded area indicates the 95% confidence interval of the mean.* ***B:*** *The means of motion correlations over X, Y and Z motion components between the two Kinect sensors are shown as smoothed violin plot histograms over participants. Means of the distributions are indicated by gray crosses and medians by black squares. Expected visibility of each joint based on visual occlusions due to either the table or the body of the participant is indicated by the background colors from white (visible in both) to dark gray (completely occluded in one of the sensors). The left-handed dyad is marked with a black dot in each distribution. Null correlations with shuffled participants' data are indicated by black horizontal lines (solid line – mean correlation over null participants, dashed lines – minimum and maximum correlations in the null distribution).* ***C and D****: Binned histogram violing plots showing the distribution of correlations of estimated angle (****C****) and linear accelerations (****D****) of the participants' head between the Kinects and IMUs. The two Kinects are plotted separately to show the dependence of each data type on the location of the Kinect sensor. The distribution of values is depicted by the vertical histograms and the means and the medians of the distributions are indicated by gray crosses and black squares, respectively. The distributions are complemented by standard box plot components (horizontal lines are min and max values and box indicates the interval between 25$^{th}$ and 75$^{th}$ percentiles). The raw data for each participant is indicated by a dot.*

## Classification of gaze behavior based on face orientation and location

The accuracy of classification of gaze behaviors based on the head location and orientation data depended on the features included in the classifier. As is seen in the confusion matrices ( **Supplementary Figure 2 A**), based on only the yaw of the face, only eye contact was predicted with a high accuracy while the other classes were confused with eye contact and each other. Similarly, over participants, the prevalence of gaze behaviors in individual subjects was well-predicted only for eye contact from yaw information alone.

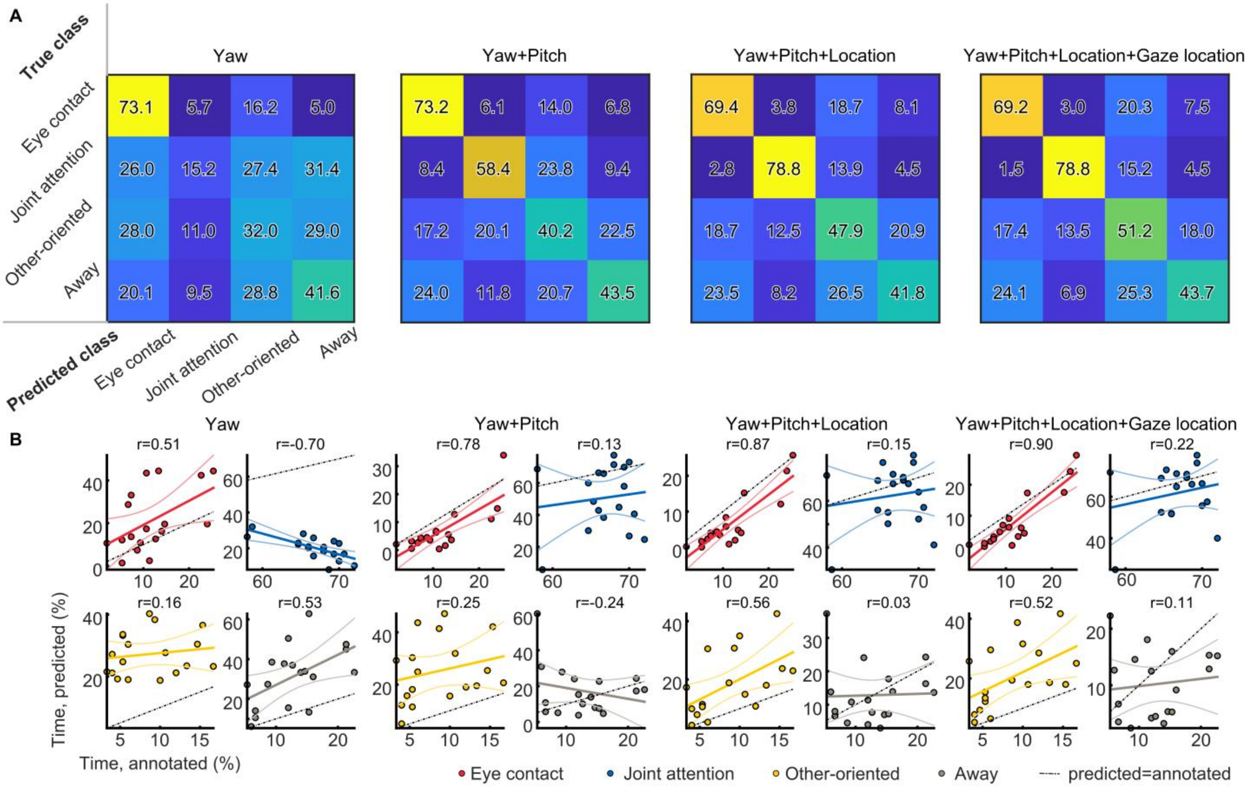


***Supplementary Figure 2: Performance of classifiers as a function of features. A*** *Confusion matrices showing accuracy for the classes on the diagonal and, on the rows, which categories each class is confused with. Based on only yaw, only eye contact is successfully recognized. When pitch and location data is added, all accuracies are increased and confusion reduces.* ***B*** *Estimated proportion of gaze behaviors based on the classifiers in full data as a function of the true proportion in the training data. Similarly to panel A, the correspondence of estimated and true prevalence of participant's gaze behaviors improves for all classes as features are added. The right-most panels show the same results as Figure 3 in the paper.*

The proportion of timepoints in all dyadic combinations of annotated behaviors (looking at partner or looking away for both individuals of a dyad) in all trials of the training data are summarized in **Supplementary Table 1**.

***Supplementary Table 1:*** *Prevalence of all combinations of gaze behaviors across all trials*

| **Partner Partner** | **Target Target** | **Partner Target** | **Away Partner** | **Away Target** | **Away Away** | **Total** |
|---|---|---|---|---|---|---|
| 11.7% | 66.8% | 8.6% | 7.8% | 2.9% | 2.1% | 100.0% |

## Relation of face and body orienting

Both face and body orientation showed significant correlation of effort and joint orienting behavior (**Supplementary Figure 3).** Faces were oriented more directly toward the communication partner as evidenced by the effect being closer to the origin (bottom-left corner, corresponding to direct joint face-to-face orienting) in the bottom panel than in the top panel. This reduced orienting of the body is particularly evident for Participant #1 of the dyads, presumably due to the way the participants are seated in the room. Generally, body and face orientations were correlated for all participants (**Supplementary Figure 4**), but the range of body angle was more limited than that of face angles particularly for Participant #1 of each dyad (displayed in green in **Supplementary Figure 4**).

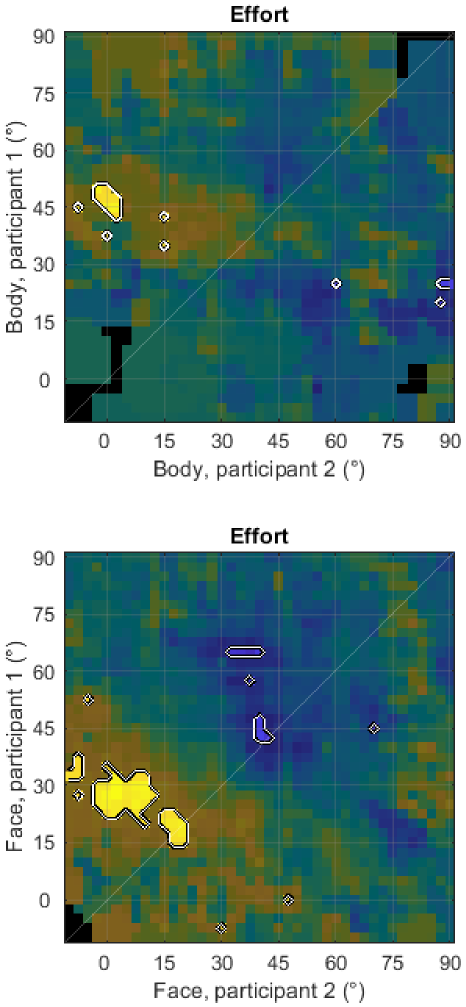


***Supplementary Figure 3: Correlation of ratings of effort and joint orientation of the body and the face with unsmoothed data.*** *The effort ratings correlate with the time participants jointly orient their bodies (top) as well as faces (bottom) toward the communication partner. The effect is shifted toward higher angles in the body results compared with the face results, particularly for participant 1.*

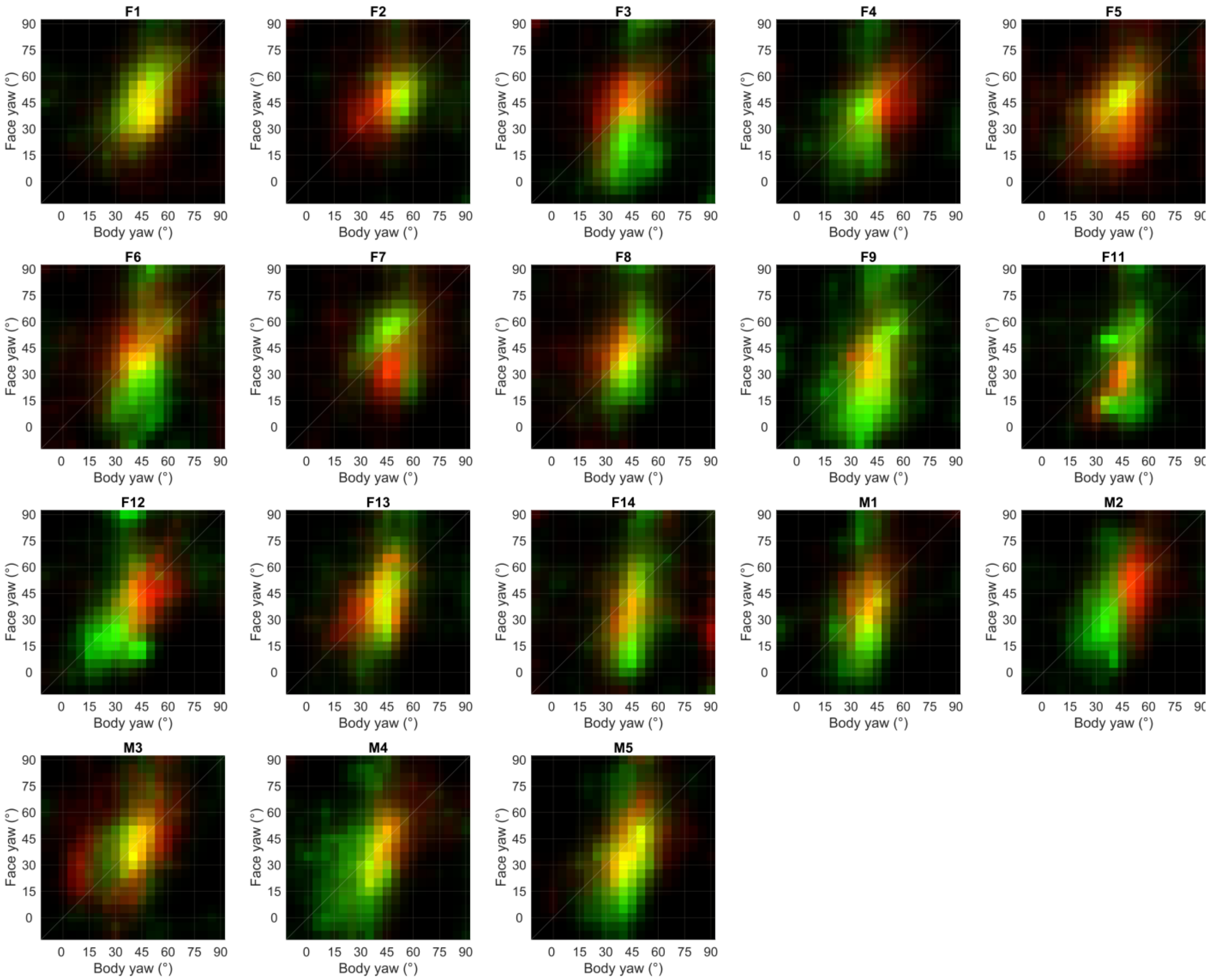


***Supplementary Figure 4: Correspondence of face and body directions.***
*Heatmaps show the face direction of all participants as a function of the body direction. Distribution of angles for participant #1 is displayed in green and participant #2 in red for each dyad. While the face directions generally show a larger range of values than body (head is turned more than the body), the movements are highly correlated for nearly all participants.*

# Supplementary discussion

## Limitations

Unexpectedly, we saw few differences in the interpersonal synchrony between conditions or as a function of behavioral ratings compared with the effects in the proxemic measures. The experimental conditions, particularly during the gameplay trials, introduce anticorrelations (or delayed synchrony) due to the turn-taking behavior, which could mask some of the differential synchrony effects across dyads. Methodologically, calculating the windowed cross-correlations requires some critical choices to be made, such as the sliding window size within which the cross-correlations are calculated and the maximum time delay at which synchrony is estimated, which might differ between conditions if particular turn-taking structure is enforced by the task. Moreover, the method produces a 2-dimensional matrix of correlation values that needs to be summarized for easier interpretation. Various measures could be used to find a representative value for the synchrony over the trials or the entire experiment. Previously, peak-picking algorithms have been suggested [1] to find the most appropriate synchrony values at a near-zero lag. However, the assumption of near-zero lag could be violated by the gameplay task, where the turn-taking introduces non-zero lags that depend on the pace of the gameplay. We did see (near) zero-lag synchrony in all conditions, but the synchrony was lower during gameplay, where we also saw synchrony peaks at non-zero lags. Because the pace of gameplay could be different between dyads, these additional peaks might be misaligned between participants and this difference in pace of turn-taking might also be of interest in some situations. In the future, it may be of interest to characterize what particular types of (delayed) synchrony and experimental conditions could reveal differences related to subjective evaluations of interactions. However, exploring the most sensitive time delays, time windows and methods for summarizing the data is beyond the scope of the current study.

In the current implementation of the tracking system, the transformations between Kinect sensors were calculated based on one of the participants. This was done due to a lack of landmarks that would be trackable by the Kinects. Additionally, although the Kinects were fixed to stands, there were sometimes small movements to the sensors between successive measurement days, which required a separate transformation matrix to be estimated for each data set separately. While the transformations were similar for all participants, using the same mean transformation for all subjects yielded results that were slightly inferior to the individual ones for the majority of the subjects. In the future, adding reflective landmarks visible to the IR camera of all Kinect sensors could improve the registration results and free the sensor placing.

While the spatial location tracking worked consistently with the two Kinects, the facial orientation tracking proved to be sensitive to errors in some participants with the current spatial layout. On average, both Kinects' facial orientation estimates were correlated with the data from the IMUs, but the results differed in their accuracy: one sensor gave very consistent estimates for 17 of the 18 participants while the second sensor's estimates were generally less accurate. To optimize the accuracy of the system, the distance to the tracked person as well as the orientation of the face in relation to the Kinect should be optimized carefully when using such setups for facial orientation tracking.

In addition to the spatial limitations, while for some dyads the temporal sampling rate stayed relatively constant at the 30 frames per second reported by the manufacturer, for other dyads the sampling rate varied considerably sometimes dropping well below the theoretical maximum. With these technical issues in mind, quality control experiments should be designed any time a new system is installed or changes have been made to an existing installation to maximize the data quality.

Finally, the correspondence of the acceleration data between the Kinects and the IMUs was relatively low compared with the orientation correspondence. This is likely caused by the higher sensitivity to short accelerations and the higher sampling rate of the IMUs compared with the Kinects, which has been reported previously [2]. In addition, the IMU data of four participants contained a sustained acceleration lasting for a several minutes apparently caused by a problem in subtracting the acceleration due to gravity by the sensor software (these accelerations were not apparent in the raw acceleration data). To remove these effects, we calculated the correlations in the time window not affected by these obvious artefactual accelerations. Checking for such artefactual accelerations or misestimations of locations in the Kinect as well as IMU data require careful quality control, which should ideally be automated in the future.

## References

1. Boker SM, Xu M, Rotondo JL, King K. 2002 Windowed cross-correlation and peak picking for the analysis of variability in the association between behavioral time series. *Psychol. Methods* **7**, 338–55.

2. Romero V, Amaral J, Fitzpatrick P, Schmidt RC, Duncan AW, Richardson MJ. 2017 Can low-cost motion-tracking systems substitute a Polhemus system when researching social motor coordination in children? *Behav. Res. Methods* **49**, 588–601.
